# The association of depression, anxiety, and stress with caring for a child with Congenital Zika Syndrome in Brazil; Results of a cross-sectional study

**DOI:** 10.1371/journal.pntd.0007768

**Published:** 2019-09-30

**Authors:** Hannah Kuper, Maria Elisabeth Lopes Moreira, Thália Velho Barreto de Araújo, Sandra Valongueiro, Silke Fernandes, Marcia Pinto, Tereza Maciel Lyra

**Affiliations:** 1 International Centre for Evidence in Disability, Clinical Research Department, London School of Hygiene & Tropical Medicine, London, United Kingdom; 2 Instituto Nacional de Saúde da Mulher, da Criança e do Adolescente Fernandes Figueira, Fundação Oswaldo Cruz, Rio de Janeiro, Brasil; 3 Postgraduate Programme in Public Health, Federal University of Pernambuco, Recife, Brazil; 4 Faculty of Public Health and Policy, London School of Hygiene & Tropical Medicine, London, United Kingdom; 5 Aggeu Magalhães Institute, FIOCRUZ/PE, Recife, Brazil; 6 Faculty of Medicine, University of Pernambuco, Recife, Brazil; Fundacao Oswaldo Cruz, BRAZIL

## Abstract

**Background:**

Zika virus (ZIKV) infection in pregnancy can cause microcephaly and a wide spectrum of severe adverse outcomes, collectively called “Congenital Zika Syndrome” (CZS). Parenting a child with disabilities can have adverse mental health impacts, but these associations have not been fully explored in the context of CZS in Brazil.

**Methodology/Principal findings:**

A cross-sectional study was undertaken in Recife and Rio de Janeiro, including 163 caregivers of a child with CZS (cases) and 324 caregivers with an unaffected child (comparison subjects), identified from existing studies. The primary caregiver, almost always the mother, was interviewed using a structured questionnaire to collect information on: depression, anxiety, and stress (Depression, Anxiety, and Stress Scale—DASS-21), social support (Medical Outcomes Study Social Support Scale—MOS-SSS), and socio-demographic data. Data was collected May 2017-January 2018. Ethical standards were adhered to throughout the research. A high proportion of mothers reported experiencing severe or extremely severe levels of depression (18%), anxiety (27%) and stress (36%). Mothers of children with CZS were more likely to experience symptoms of depression, anxiety andstress, compared to mothers of comparison children. These associations were more apparent among mothers living in Rio de Janeiro. These differences were reduced after adjustment for socio-economic status and social support. Among mothers of children with CZS, low social support was linked to higher levels of depression, anxiety and stress, but there was no association with socio-economic status.

**Conclusions/Significance:**

Depression, anxiety and stress were very common among mothers of young children in Brazil, regardless of whether they were parenting a child with disabilities. Mothers of children with CZS may be particularly vulnerable to poor mental health, and this association may be buffered through better social support.

## Author summary

The 2015 Zika epidemic in Brazil gained international attention with the birth of thousands of babies with severe adverse outcomes, collectively called “Congenital Zika Syndrome” (CZS). Parenting a child with disabilities can be extremely stressful, and in other settings is linked to depression, anxiety and other mental health conditions. However, these associations have not been fully explored in the context of CZS in Brazil. We conducted a cross-sectional study in Rio de Janeiro and Recife, two cities in Brazil. We identified 163 caregivers (mostly the mother) of a child with CZS, and 324 caregivers of children of similar ages, but without obvious disabilities. The caregivers were asked a series of questions to gauge whether they were experiencing depression, anxiety or stress. We found that a high proportion of the mothers reported experiencing severe or extremely severe levels of depression (18%), anxiety (27%) and stress (36%). Levels of depression and anxiety were even higher for mothers of children with CZS compared to mothers of unaffected children, especially for those mothers with low social support and those living in Rio de Janeiro.

## Introduction

Brazil hit international headlines in late 2015 with the birth of thousands of babies with microcephaly, which was soon linked to congenital infection with the Zika Virus (ZIKV). It was quickly apparent that babies born after congenital ZIKV infection experience a range of severe conditions beyond microcephaly alone, which is now collectively called “Congenital Zika Syndrome” (CZS). [1] Phenotypic characterization is still ongoing, but to date CZS is indicated by: microcephaly, other patterns of brain damage (including subcortical calcifications), damage to the back of the eye, congenital contractures, and hypertonia restricting body movement soon after birth. Children with CZS therefore have multiple and broad-ranging impairments, and as a result have complex care needs, which lie mostly with parents, in particular the mother. By early 2018, 3,149 cases of CZS were registered in Brazil, with many more potential cases not officially confirmed. [2]

Evidence is growing that carers of children with disabilities are more likely to experience depression, anxiety and stress, [3–5] and this may make it more difficult to meet the care needs of their child. Mental health impacts arise through different potential pathways. It may be distressing and overwhelming to meet the complex needs of a child with disabilities, which will continue for the lifetime of the child. Prevalence of depression and anxiety may therefore be particularly high among the carers of children with greater functional difficulties. [3, 4] Parents of children with disabilities may also experience negative attitudes and discrimination, and consequent social isolation, including marital breakdown, [6, 7] which can contribute to poor mental health. Caring for a severely disabled child often has cost implications, both direct costs (e.g. treatment) and indirect costs (e.g. foregone wages as the mother does not return to work), [8] creating financial problems, [7] and thereby increasing the risk of depression, anxiety and stress. [9] On the positive side, good social support and access to adequate resources may buffer the negative mental health impacts for carers.[10]

Carers of children with CZS may be particularly vulnerable to poor mental health. [11] These children experience severe impairments, with high healthcare needs. Affected children may be highly irritable and difficult to care for—as one mother described: *“For every ten minutes of sleep*, *she cries for an hour”*.[12] Another source of distress in relation to CZS is the uncertainty about the long-term trajectory of the condition, given its newness, and a lack of specialized professional knowledge. Families of children with CZS are also on average poorer and therefore may be less resilient to these challenges. [13] However, limited evidence is available on the impacts of CZS on the mental health of carers, despite the strong case for why a link is likely. One small study, conducted in Sergipe State in Brazil compared levels of anxiety and depression in the first 24 hours after birth between 9 mothers of newborns with microcephaly and twenty mothers with healthy newborns. [14] Mothers of babies with microcephaly had lower scores in the psychological domain of quality of life than those with healthy newborns, but there was no difference in anxiety (which was high in both groups) or depression. After ten months of follow-up, the mothers of children with CZS reported high levels of anxiety and low quality of life.[15] However, these results must be interpreted with caution due to the very small sample size, and other relevant studies are lacking.

In order to address these gaps in the literature, the current study aimed to explore the association of having a child affected by CZS with depression, anxiety, and stress, and to assess whether these relationships are buffered by social support and socio-economic status.

## Methods

### Overview and setting

The methods of the study have been described in full elsewhere. [16] Briefly, a cross-sectional study was undertaken to explore the differences between caregivers with a child affected by CZS to those of typically developing children, in terms of social and economic indicators. Two contrasting sites were selected. The first was metropolitan areas in Recife, in the State of Pernambuco in Northeast Brazil, which had a high number of suspected and confirmed cases of CZS. [2] The second site was Rio de Janeiro City, where symptomatic ZIKV was less prevalent and reports of CZS far lower.

### Sample size

We aimed to recruit 100 cases of children with CZS and 100 comparison subjects without CZS per setting, which would provide the power to detect an OR of 2.6 in each site for the association between depression and CZS, assuming 95% confidence, 80% power and a prevalence of depression of 15% in unaffected caregivers. [17] Across the two samples (i.e. 200 cases and 200 comparison subjects), the sample size would be adequate to detect an OR of 2.05 for the same association.

### Recruitment of cases and comparison subjects

In Recife, the source of most of the cases and all the comparison subjects was an existing case-control study, initiated in January 2016 to identify causes of CZS. [18] Cases were children born with microcephaly (head circumferences < 2 SD than the mean) in eight public maternity hospitals in Recife. Controls were children born in the same hospitals, but without microcephaly and without apparent neurological or other health problems (determined from transfontanellar ultrasonography, and through physical examination by the study neonatologist), with both examinations performed soon after birth. Controls were matched to cases on the basis of expected date of delivery and place of mother’s residence (by Health Region). At follow-up, caregivers of controls were asked whether there were any developmental delays (using the Denver test) and if the response was positive, the control child was excluded from the study and referred for further investigation. Additional cases with CZS were identified from an ongoing “cohort of children”. These children were identified as potentially having CZS from those born to a cohort of pregnant women who presented with a rash (a common symptom of ZIKV infection), and from outpatient clinics of children with CZS. Suspect cases were examined by a pool of specialists to confirm CZS. At follow-up, the participants were are classified as “cases” and “comparison subjects” (rather than cases and controls), to reflect the fact that we were assessing cross-sectional association of mental health conditions and CZS, rather than identifying exposures which may be aetiologically linked to CZS.

In Rio de Janeiro, the source of the cases and comparison subjects was the Vertical Exposure to Zika Virus and Its Consequences for Child Neurodevelopment: Cohort Study in Fiocruz/IFF (ClinicalTrials.gov Identifier: NCT03255369). Cases were children born to mothers known to be ZIKV positive, who had microcephaly. Comparison subjects were born to mothers without a history of symptoms and without developmental delay, as shown by: 1) a composite Bayley Score ≥85 conducted between 6 and 36 months following the recommended guidelines and/or 2) assessment by two paediatricians based on the child’s medical records. [19]

### Data collection

The primary caregiver, usually the mother, was interviewed using a structured questionnaire.

The Depression, Anxiety, and Stress Scale (DASS-21) was used to assess symptoms of depression, anxiety, and stress among caregivers. [20] It is a 21-item questionnaire with a four-point (0–3) answer scale that focuses on the extent participants had experienced certain symptoms over the previous week. Items are arranged into three subscales (depression, anxiety and stress), with seven items for each subscale. DASS-21 is a reliable tool to assess psychological distress and has been adapted and validated for Brazilian Portuguese. [21] The Medical Outcomes Study Social Support Scale (MOS-SSS) was used to measure social support. It is a 19-item questionnaire with each item scored on a Likert scale of 1 to 5, which includes five scales covering different aspects of social support (affection, positive social interaction, emotional, informational, and material). It has also been validated for Brazilian Portuguese. [22] Data was also collected on: the parents’ socio-demographic characteristics (e.g. asset ownership, parental marital status).

Data collection was undertaken between May 2017 and January, 2018. In Recife, the caregivers were interviewed in their homes, at the Primary Health Centre or occasionally, in their workplace. In Rio de Janeiro the interview was undertaken in person at attendance at The Fernandes Figueira Institute (IFF). In Recife, four quantitative interviewers were included (all female) and three were included in Rio de Janeiro (two female one male), all had a degree in social science or public health.

### Quantitative data analysis

Variables were created from the standardized questionnaires. For DASS-21, sub-scales were calculated for Depression, Anxiety, and Stress Scale with each subclass’s score equal to the sum of seven corresponding questions. The sum scores were multiplied by 2 in order to match the original scale score in DASS-42 so that each subscale score ranges from 0 to 42. [20] Categories were created for:

- Depression (normal: <9; mild: 10–13, moderate: 14–20, severe: 21–27, extremely severe: >28).
- Anxiety (normal: <7; mild: 8–9, moderate: 10–14, severe: 15–19, extremely severe: >20).
- Stress (normal: <14; mild: 15–18, moderate: 19–25, severe: 26–33, extremely severe: >= 34).

For MOS-SSS, an overall social support index was calculated, ranging from 0 to 100, with higher score indicating better availability of social support. In addition, four separate social support functional subscales were generated measuring: 1) emotional/informational social support, 2) tangible social support, 3) affectionate social support, and 4) positive social interaction.

Socio-demographic characteristics were regrouped into categories, to reflect the distribution of the data among the participants (groupings shown in Table 1). The Social-economic strata were estimated following the Brazilian Criteria, based on household assets from a pre-defined list, head of household education as well as the household’s access to piped water and a paved street. The household income estimates in each strata are approximations based on the 2013 National Household Sample Survey. [23]

**Table 1:** Baseline characteristics of mothers with a child with microcephaly and comparison mothers.

	Recife (n = 193)			Rio de Janeiro (n = 237)		
	Cases (n = 81)	Comparison subjects (n = 112)	X^2^ p-value	Cases (n = 82)	Comparison subjects (n = 155)	X^2^ p-value
**Age group mother**						
<20 years	13%	10%	0.27	11%	4%	0.008
20–29 years	48%	54%		53%	41%	
30–39 years	33%	34%		34%	44%	
40+years	8%	2%		3%	11%	
Marital status						
Married	33%	32%	0.85	28%	45%	0.03
Cohabiting	38%	36%		43%	37%	
Other	28%	32%		29%	19%	
Age of child						
<12 months	10%	27%	<0.001	50%	28%	0.004
12 months-<18months	14%	65%		35%	54%	
18 months+	77%	8%		15%	18%	
Gender of child						
Male	43%	55%	0.10	50%	47%	0.67
Female	57%	45%		50%	53%	
Education mother						
None-Junior high completed	57%	49%	0.43	37%	25%	0.01
High school completed	43%	50%		59%	57%	
University degree completed	0%	1%		5%	17%	
Economic strata (reference average income per strata)						
A-B (R$ 4427–20273)	4%	4%	0.48	17%	32%	0.03
C1 (R$ 2409)	16%	11%		23%	27%	
C2 (R$1446)	30%	39%		34%	27%	
D-E (R$ 640)	51%	46%		26%	14%	
Number of people living in household (incl. child)						
≤4	40%	32%	0.57	43%	40%	0.49
4	27%	31%		27%	34%	
≥5	33%	37%		30%	26%	

A chi-square test was used to compare the socio-demographic characteristics of cases and comparison subjects, to identify potential confounders of the association of CZS with mental health outcomes. The mean depression, anxiety and stress levels were compared for carers of children with CZS and comparison subjects using a Wilcoxon rank sum test.

Multivariable logistic regression analyses were undertaken using Stata (version 15) to compare the odds of study outcomes (i.e. maternal depression, anxiety and stress) among carers of children with CZS to those of unaffected children, adjusted for age, socio-economic status (SES) variables and location (Rio de Janeiro/Recife). We tested associations for effect-modification by, in turn, location, social support and economic status. There were few missing data, and analyses were conducted for participants where complete data for relevant indicators were available.

### Ethics statement

Ethical approval for the full study was received from LSHTM and the Fiocruz ethics committee (CAAE 60682516.2.1001.5269). The original case-control study in Recife was approved by the Research Ethics Committees of the Pan American Health Organization (PAHO-2015-12-0075) and Fiocruz Pernambuco (CAAE: 51849215.9.0000.5190) and the Cohort study in Rio de Janeiro was approved by the IFF Ethics Committee (CAAE 52675616.0.0000.5192). The “Cohort of children” in Recife was approved by the ethics Committee of the Oswaldo Cruz University Hospital, University of Pernambuco (CAAE: 52803316.8.0000.5192). All interviewees were adults and all provided written informed consent.

## Results

The study included 81 cases and 112 comparison subjects from Recife, and 82 cases and 155 comparison subjects from Rio de Janeiro (Table 1). Among those interviewed, 97.5% were the mother, 1.4% the father and 1.0% the grandmother, and so the carer is referred to as the “mother” throughout for convenience. In both locations, mothers of cases were significantly younger than mothers of comparison subjects. Furthermore, in Recife the case children were older than the comparison children, whereas in Rio de Janeiro the reverse was true. In Rio de Janeiro, the case mother was less likely to be living in a union (71% versus 81% for comparison subjects), was less likely to be educated beyond the primary level (64% versus 74% for comparison subjects), and was in a lower economic stratum as compared to the comparison subjects. These differences were not apparent in the Recife sample, and in both locations the cases and comparison subjects were similar in terms of the number of people living in the household and gender of the child.

Median depression (p = 0.01), anxiety (p = 0.03) and stress scores (p = 0.05) were higher among cases than comparison subjects (Table 2). After stratification by location it was apparent that in Rio de Janeiro, scores were significantly higher for cases than comparison subjects for depression (p = 0.02), anxiety (p = 0.006) and stress (p = 0.02), while no differences were apparent in Recife between cases in comparison subjects.

**Table 2:** Comparison of depression, anxiety and stress scores among cases and comparison subjects, by location.

	Location		N	Median	Inter-quartile range	Wilcoxon rank-sum p-value
Depression score	Recife	Cases	81	8	4–20	0.28
		Comparisons	112	6	2–18	
	Rio de Janeiro	Cases	82	10	2–20	0.02
		Comparisons	155	6	2–18	
	All	Cases	163	10	2–20	0.01
		Comparisons	267	6	2–16	
Anxiety score	Recife	Cases	81	8	2–18	0.92
		Comparisons	112	6	2–18	
	Rio de Janeiro	Cases	82	8	4–18	0.006
		Comparisons	155	4	0–12	
	All	Cases	163	8	2–18	0.03
		Comparisons	267	6	0–16	
Stress score	Recife	Cases	81	16	10–32	0.75
		Comparisons	112	18	8–30	
	Rio de Janeiro	Cases	82	22	12–34	0.02
		Comparisons	155	18	8–26	
	All	Cases	163	20	10–34	0.05
		Comparisons	267	18	8–28	

Overall, a high proportion of mothers (across cases and comparison subjects) reported experiencing severe or extremely severe levels of depression (18%), anxiety (27%) and stress (36%) (Table 3). Unadjusted analyses showed that mothers of children with CZS showed higher levels of “any” depression (OR = 1.5, 95% CI = 1.0–2.2) or “any” anxiety (1.4, 1.0–2.1) compared to comparison subjects. However, these associations were not apparent after adjustment for socio-economic status and social support. There did not appear to be a trend in this association by severity of depression or anxiety. No differences in stress levels were identified between cases and comparison subjects.

**Table 3:** Depression, anxiety and stress in mothers with a child with CZS, and comparison subjects.

	Cases (n = 163)	Comparison subjects (n = 267)	Unadjusted OR (95% CI)	OR adjusted for maternal age and child’s age (95% CI)	OR adjusted for maternal age, child’s age and socio-economic status (95% CI)	OR adjusted for maternal age, child’s age and socio-economic status and overall social support (95% CI)
	%	%				
Depression						
Normal	49%	58%	1.0	1.0	1.0	1.0
Mild/moderate	30%	25%	1.4 (0.9–2.3)	1.5 (0.9–2.6)	1.5 (0.9–2.5)	1.3 (0.8–2.3)
Severe/ extremely severe	21%	16%	1.5 (0.9–2.5)	1.5 (0.8–2.7)	1.4 (0.8–2.5)	1.3 (0.7–2.4)
Any depression (≥mild)	51%	42%	1.5 (1.0–2.2)	1.5 (1.0–2.3)	1.4 (0.9–2.2)	1.3 (0.8–2.0)
Anxiety						
Normal	47%	56%	1.0	1.0	1.0	1.0
Mild/moderate	22%	19%	1.4 (0.8–2.3)	1.1 (0.6–2.0)	1.2 (0.6–2.1)	1.1 (0.6–1.9)
Severe/ extremely severe	31%	25%	1.5 (0.9–2.4)	1.5 (0.9–2.4)	1.4 (0.8–2.3)	1.3 (0.7–2.2)
Any anxiety (≥mild)	53%	44%	1.4 (1.0–2.1)	1.3 (0.8–2.0)	1.2 (0.8–1.9)	1.1 (0.7–1.8)
Stress						
Normal	40%	43%	1.0	1.0	1.0	1.0
Mild/moderate	19%	24%	0.9 (0.5–1.4)	1.1 (0.6–2.0)	1.2 (0.6–2.1)	1.1 (0.6–2.1)
Severe/ extremely severe	41%	32%	1.4 (0.9–2.2)	1.5 (0.9–2.4)	1.4 (0.9–2.4)	1.3 (0.7–2.1)
Any stress (≥mild)	60%	57%	1.2 (0.8–1.7)	1.3 (0.8–2.0)	1.3 (0.8–2.0)	1.2 (0.7–1.9)

The association between depression, anxiety and stress (in turn) and components of social support were investigated among the mothers of children with CZS (Table 4). Mothers of children with CZS were more likely to experience symptoms of depression if they had low levels of social support, and this was consistent across all domains of social support. For anxiety and stress, this pattern was apparent in relation to overall social support, positive social interaction, and emotional/informational support, but not for the other domains of social support.

**Table 4:** Association of social support with depression, anxiety and stress among mothers of child with CZS.

Component of social support	Any depression (N = 83) versus none (N = 80) OR (95%CI)	Any anxiety (N = 87) versus none (N = 76) OR (95%CI)	Any stress (N = 98) versus none (N = 65) OR (95%CI)
Emotional/informational support			
All the time	1.0	1.0	1.0
Most of the time	2.3 (1.0–5.5)	2.5 (1.1–6.0)	2.4 (1.0–5.8)
Some, a little, none of the time	6.4 (2.9–14.0)	2.9 (1.4–6.0)	4.2 (1.9–9.1)
Tangible support			
All the time	1.0	1.0	1.0
Most of the time	1.5 (0.7–3.2)	1.3 (0.6–3.0)	1.1 (0.5–2.5)
Some, a little, none of the time	2.6 (1.3–5.5)	1.6 (0.8–3.2)	1.2 (0.6–2.5)
Affectionate support			
All the time	1.0	1.0	1.0
Most of the time	2.0 (0.9–4.5)	1.9 (0.8–4.3)	1.6 (0.7–3.7)
Some, a little, none of the time	3.1 (1.2–7.7)	1.8 (0.8–4.4)	1.9 (0.8–4.7)
Positive social interaction			
All the time	1.0	1.0	1.0
Most of the time	3.1 (1.4–7.1)	1.6 (0.7–3.7)	2.0 (0.9–4.4)
Some, a little, none of the time	6.2 (2.8–13.5)	5.6 (2.5–12.4)	5.3 (2.3–12.2)
Overall social support			
All the time	1.0	1.0	1.0
Most of the time	2.6 (1.2–5.7)	1.6 (0.7–3.3)	1.7 (0.8–3.6)
Some, a little, none of the time	6.9 (2.9–16.4)	3.1 (1.4–6.9)	3.6 (1.5–8.4)

The association between parenting a child with CZS and depression, anxiety and stress (in turn) was stratified by socio-economic status, social support and location (Table 5). No significant effect modification was detected for any of these comparisons Parents of children with CZS living in Rio de Janeiro appeared more likely to experience depression, anxiety and stress than parents of children with CZS living in Recife, but the p-value for interaction did not reach statistical significance.

**Table 5:** The association of depression, anxiety and stress with parenting a child with CZS, stratified by SES, social support and location.

	Socio-economic status			Social support			Location		
	Low SES (C2-E) (n = 274)	High SES (A-C1) (n = 156)	P for interaction	Low social support (≤some of the time) (n = 73)	High social support (≥most of the time) (n = 355)	P for interaction	Recife (n = 193)	Rio de Janeiro (n = 237)	P for interaction
	OR (95% CI)	OR (95% CI)		OR (95% CI)	OR (95% CI)		OR (95% CI)	OR (95% CI)	
Depression									
None	1.0	1.0		1.0	1.0		1.0	1.0	
Any	1.5 (0.9–2.4)	1.2 (0.6–2.4)	0.60	1.9 (0.7–5.3)	1.1 (0.7–1.8)	0.40	1.1 (0.6–1.9)	1.9 (1.1–3.3)	0.14
Anxiety									
None	1.0	1.0		1.0	1.0		1.0	1.0	
Any	1.5 (0.9–2.4)	1.3 (0.7–2.6)	0.82	1.3 (0.5–3.4)	1.3 (0.8–2.0)	0.99	1.2 (0.7–2.2)	1.6 (0.9–2.8)	0.51
Stress									
None	1.0	1.0		1.0	1.0		1.0	1.0	
Any	1.1 (0.7–1.8)	1.2 (0.6–2.3)	0.93	1.4 (0.5–4.0)	1.0 (0.6–1.5)	0.54	0.9 (0.5–1.6)	1.5 (0.9–2.6)	0.22

## Discussion

This cross-sectional study compared the mental health of mothers of children with CZS to those with developmentally typical children in Brazil. The study found that a high proportion of mothers reported experiencing severe or extremely severe levels of depression, anxiety and stress, even among mothers of children without apparent disabilities. Mothers of children with CZS were more likely to experience symptoms of depression and anxiety, but not stress, and these differences disappeared after adjustment for socio-economic status and social support. Among mothers of children with CZS, low social support was linked to higher levels of depression, anxiety and stress. After stratification by location, anxiety and depression were associated with parenting a child with CZS in Rio de Janeiro, but not in Recife.

Previous studies have consistently demonstrated an association between parenting a child with neurodevelopmental disabilities and the experiences of depression, anxiety and stress.[3–5, 9] We can speculate on the reason for the disparity with the results of the current study, where these associations were less strong and consistent. ZIKV was reported about widely in the media in Brazil, and so there was a high awareness of the condition, and potentially less stigma experienced by parents than occurs for other types of childhood disability. The Brazilian government committed to the support of children with CZS and their families, and consequently many families may be able to access to health services and social benefits, reducing financial stress. There are also some social support structures in Brazil for parents of CZS, ranging from informal Whatsapp groups to established NGOs, which may help to buffer the negative mental health impacts of caring for a child with disabilities. Furthermore, poor mental health appeared to be extremely common among the comparison subjects, making it more difficult to detect a difference with cases, perhaps because of the pervasive social issues experienced in Brazil currently. The negative mental health impacts of having a child with CZS was more pronounced in parents living in Rio de Janeiro than in Recife. A potential explanation may be that the comparison mothers in Rio de Janeiro were generally older, more likely to be living in a union, and had higher levels of education and socio-economic status than the mothers in the other groups, which may influence the lower levels of depression and anxiety. In Recife, the cases and comparison subjectswere matched by location, and as women living in poverty and vulnerable situations are more likely to experience mental health concerns, differences between cases and comparison subjectswould have been reduced.

The mental health of carers of children with disabilities remains an important concern. However, in this context the high prevalence of depression, anxiety and stress among mothers of unaffected children was disturbing and needs urgent attention. Depression, anxiety and stress reduce the quality of life of those affected and are also linked to poorer physical health and social functioning. [24, 25] Parental depression can also result in negative parenting behaviours and reduced interaction with the child, so that the child may not receive the stimulation needed to thrive,[24, 25] which is particularly of concern in the case of children with disabilities, who are already facing challenges in their development. It is therefore important to identify the mental health needs of parents of children with disabilities, so that appropriate interventions can be implemented. A recent Cochrane review reported that psychological therapies improved parental mental health for parents of children with cancer post- treatment, [26] although evidence was lacking of positive impacts for parents of children with other chronic conditions. Improving social support may also help to buffer the negative impacts of caring on mental health, for instance, through establishing parent support groups, offering relationship counselling, or developing informal support networks (e.g. Whatsapp groups). [10, 11, 27] Improving mental health and social support of carers, may also help to improve adaptive parenting behavior, [26] and thereby benefit the child. Currently, more research is needed on which interventions work to improve mental health and social support of carers, particularly in low resource settings.

There are important strengths and limitations of the study, which must be taken into account when considering the findings. This was a relatively large study, conducted across two contrasting sites, and including comprehensive questionnaires using validated scales for depression, anxiety, stress and social support. This study also fills an important gap, as currently robust quantitative data on the mental health impacts of carers of children with CZS is lacking. In terms of limitations, clinical diagnosis of mental health conditions in the parents was lacking, and so we relied on screening questionnaires for assessing symptoms. The number of cases recruited was smaller than planned (though the number of comparison subjectswas higher), and the association between depression and CZS was weaker than anticipated, and so some of the analyses may have been under-powered. In particular, we were under-powered to identify effect modifiers.

### Conclusions

This study found that depression, anxiety and stress were very common among mothers of young children in Brazil. Mothers of children with CZS may be particularly vulnerable to poor mental health, and this impact may be buffered by improving social support.

## Supporting information

S1 Checklist Strobe checklist. (DOC)
